# Lambda-Display: A Powerful Tool for Antigen Discovery

**DOI:** 10.3390/molecules16043089

**Published:** 2011-04-13

**Authors:** Elisa Beghetto, Nicola Gargano

**Affiliations:** Sigma-tau Industrie Farmaceutiche Riunite S.p.A., Rome, Italy; E-Mail: elisa.beghetto@sigma-tau.it

**Keywords:** bacteriophage lambda, phage display, antigen discovery, diagnostic assay, vaccine

## Abstract

Since its introduction in 1985, phage display technology has been successfully used in projects aimed at deciphering biological processes and isolating molecules of practical value in several applications. Bacteriophage lambda, representing a classical molecular cloning and expression system has also been exploited for generating large combinatorial libraries of small peptides and protein domains exposed on its capsid. More recently, lambda display has been consistently and successfully employed for domain mapping, antigen discovery and protein interaction studies or, more generally, in functional genomics. We show here the results obtained by the use of large libraries of cDNA and genomic DNA for the molecular dissection of the human B-cell response against complex pathogens, including protozoan parasites, bacteria and viruses. Moreover, by reviewing the experimental work performed in recent investigations we illustrate the potential of lambda display in the diagnostics field and for identifying antigens useful as targets for vaccine development.

## 1. Overview of Display Technologies

Display technologies allow the exploration of large repertoires of biological molecules by the use of efficient selection and rapid characterization procedures. Biological display utilizes the cellular biosynthesis machinery to assemble biopolymers, the sequence of which ultimately specifies structures with distinct properties. The most extensively used systems utilize large repertoires (libraries) of polypeptides and proteins to be challenged with protein targets (*i.e.*, antibody domains, enzymes, signal transduction proteins, cell surface receptors) or non-protein targets (*i.e.*, carbohydrates, polymers or other surface materials) in screening procedures for the isolation of specific interacting partners [1,2].

So far, two different display systems have been developed: *(i) cell-free* systems, based on libraries of polypeptide–DNA/RNA complexes, in which each polypeptide is physically bound to its nucleic acid-coding sequence [3,4,5,6]; and *(ii) cell-based* systems, based on libraries of natural or synthetic nucleotide sequences which are translated and assembled in host cells [7,8,9,10,11,12,13]. Among them, the most common display format is the bacteriophage display, called phage display, representing a viable and efficient option for the generation of very large repertoires (up to 10^12^–10^13^ different particles) obtained at low cost by simple methods of molecular biology.

Since its introduction in 1985 [14], phage display has demonstrated to be effective for producing large libraries of polypeptides and efficiently isolating molecules with a given function. Also, it has been employed for selecting antigens [15,16,17,18] and characterizing epitopes [19,20,21,22] and antibodies [23], for investigating protein-protein interactions [24,25] and for enhancing affinity in protein-ligand interaction [26,27].

In phage display systems, nucleotide sequence repertoires such as mRNAs, cDNAs, genomic DNA fragments, and synthetic oligonucleotides are cloned into bacteriophage genomes with a specific phenotype/genotype linkage: each virus contains the genetic information for the ectopic element displayed on its capsid. In such a way the displayed recombinant molecule can interact with the corresponding target allowing the isolation of specific phage clones from pools of billions of distinct recombinant viruses. The genotype-phenotype linkage also allows a rapid determination of the amino acid sequence corresponding to the specific binding polypeptide through the direct sequencing of DNA inserts from the selected phage particle. Although other molecular methods such as conventional lambda expression libraries (*i.e.*, λgt11, where ectopic polypeptides are expressed in the cytoplasm of the host cell but they are not exposed onto bacteriophage’s surface), two-hybrid system (functional co-expression of polypeptides in yeast cells) and proteomics have proven to be successful in identifying interacting partners, phage display reserves important advantages, particularly in the areas of protein engineering and functional genomics, due to the enormous diversity of sequences that can be displayed on bacteriophage surfaces.

Developed first, phage display exploited the single-stranded DNA filamentous bacteriophage fd, M13 or related phagemid systems. In such systems, foreign peptides are displayed on the phage capsid as N-terminal fusions to pVIII or pIII coat proteins, while the assembly of filamentous particles occurs between the inner and outer membranes of *Escherichia coli* without causing lysis or loss of viability of the host cell. Since there are about 2,700 copies of pVIII per single virion, while only five copies of pIII are located at one end of the particle, highly multivalent display libraries can be produced by generating fusions to pVIII, usually achieved via two-gene systems that result in phage particles displaying a mixture of wild-type and recombinant proteins. 

An essential requirement for proper display of either a peptide or protein is that the capsid fusion itself does not interfere with phage morphogenesis. Membrane assembly of pIII [28] and pVIII [29] may be blocked by insertion of amino acids with positive charges close to the signal sequence cleavage site. This is because the positive charges reduce a proper pIII/pVIII insertion into the *E. coli* inner membrane, thus blocking the assembly and extrusion of filamentous phage particles. Consequently, many cytoplasmic proteins cannot be translocated across bacterial membranes and correctly displayed as fusion proteins on the bacteriophage surface. In addition, large protein domains fused to pVIII often disturb the process of protein transport and assembly of filamentous phage particles. In contrast, pIII is less sensitive to the size of foreign peptides, but is present in much lower number of copies than pVIII (five copies per particle), which dramatically reduces the avidity contribution in the ligand binding (the sum of multiple affinities, for example when a polyvalent antibody binds to a polyvalent antigen). This highly limits the selection efficiency of ligands which are available at low concentration or are present in complex mixtures (as in the case of biological fluids, such as antibodies in serum samples). 

In general, filamentous phage display represents a powerful tool but it also has significant drawbacks, especially when used for representing complex repertoires from natural sources, such as cDNA and genomic DNA, where it is likely that a sizeable fraction of the repertoire will not be represented in the library. This is because only those recombinant proteins able to pass through the inner bacterial membrane, still maintaining their folding in the oxidizing environment of the periplasmic space, will be correctly displayed. As a matter of fact, few reports have been published on successful display of cDNA libraries on filamentous phages [30,31]. 

One alternative to avoid the limitations imposed by secretion would be the use of phage vectors directing capsid formation in the cytoplasm, rather than by extrusion through the cellular membrane. Accordingly, recent alternative display systems exploited three lytic bacteriophages, characterized by very different life cycles, but sharing the common property of being assembled in the cytoplasm and then released by cell lysis: lambda [32,33,34,35,36] T7 [37] and T4 [38,39]. In these bacteriophages, the display of fusion proteins does not depend on their ability of being translocated across the bacterial membrane. Overall, the filamentous phage display is suited for the expression of secreted proteins, while the lambda phage and other lytic bacteriophages (T7 and T4) are excellent for the display of cytoplasmic proteins.

## 2. The Lambda Bacteriophage

**Figure 1:**
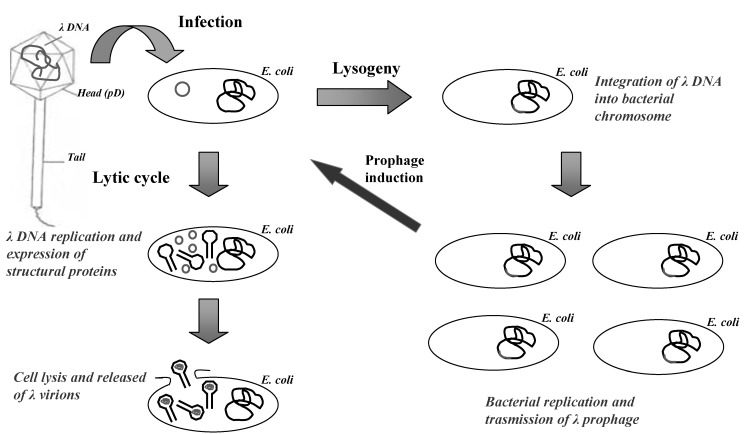
The life cycle of lambda bacteriophage.

Lambda is a temperate bacteriophage of *E. coli*, characterized by a double-stranded DNA genome of 48,502 bp in length. Inside the bacteriophage head the viral genome is packaged as a unique double-stranded linear molecule with two single-stranded protruding terminals of 12 nucleotides (cohesive ends). When lambda infects the bacterium, the linear DNA molecule is injected into the host rapidly forming a circular molecule that serves as a transcription template during the early uncommitted phase of infection (Figure 1).

The lambda genome is organized into three functionally related gene clusters: *(i*) the left-hand region, including genes responsible for packaging and assembling the DNA genome into bacteriophage head; *(ii*) the central region, including genes involved in establishment and maintenance of lysogeny, and genes not essential for lytic growth (useful for cloning of DNA inserts); *(iii*) the right-hand region, containing genes which are essential for DNA replication and lysis of infected bacteria.

During lysogenic state, the lambda genome is stably integrated into the bacterial chromosome and is replicated as a part of the host genome and transmitted to the bacterial progeny. During lytic cycle, the circular DNA directs the synthesis of proteins required for viral replication, assembly of bacteriophage particles and cell lysis. In this phase, lambda genome is replicates bi-directionally by a “rolling circle” mechanism, producing a long linear concatemeric substrate for DNA packaging. After 40–45 min the lytic cycle is concluded by bacterial lysis with the production of about 100 viral particles per infected bacterium.

The viral particle is constituted by an icosahedral head attached to a flexible helical tail. The mature head consists of 415 and 405–420 copies of the major capsid proteins gpE and gpD, respectively [40]. During phage particle generation, an empty capsid, called the pro-head, is first assembled. The assembly is then accompanied by the expansion of the capsid and by the addition of gpD [41]. Subsequently, lambda DNA is inserted and packaged into the pro-head. Finally, the tubular part of the flexible tail is assembled, consisting of 32 disks each containing six subunits of the major tail protein gpV, and added to the mature head [41].

## 3. Lambda Vectors for Display Applications

Lambda bacteriophage has been demonstrated to be the system of choice to display complex cDNA libraries, and both gpV and gpD proteins have been used as fusion partners. Libraries with a complexity of 10^7^–10^8^ independent clones can be easily constructed using efficient *in vitro* packaging extracts. 

The pioneering vector for displaying foreign proteins onto lambda capsid employed the gpV tail protein as the fusion partner. In 1994, Maruyama and co-workers engineered a lambda vector (λfoo) which allows the expression of foreign polypeptides as fusions to the C-terminus of a truncated gpV protein by replacing the last 70 amino acids of the tail protein [33]. However, though these fusion constructs have been used efficiently for panning the libraries with specific antibodies, the gpV fusion system showed some limitation, including a low display level (*i.e.*, few fusion products per phage particle) with the consequent low yield of phage recovery after affinity purification. On the other hand, the low display level of the gpV fusion protein has an advantage since it can distinguish low-affinity and high-affinity binders, and one can isolate only lambda phage clones with high affinity for bates.

To increase the display level of the fusion products, and hence the phage recovery in screening procedures, the head decoration protein gpD has been explored as fusion partner of foreign proteins. The abundance of gpD (405–420 copies per capsid), a small capsid protein (11.4 kDa) essential for phage morphogenesis, makes this coat protein the ideal fusion partner. Accordingly, it has been demonstrated that gpD can tolerate both amino- and carboxyl-terminal insertions of peptides and protein domains accessible for ligand interaction without interfering in phage replication and assembly of infective bacteriophages [34,36]. 

Although it has been reported that foreign polypeptides can be displayed on gpD as “full display” at very high density (up to 90% of the gpD copies) [42], large protein domains which might interfere with phage morphogenesis are more commonly displayed using a two-gene system, where both wild-type gpD and recombinant D-fusion genes are co-packaged into lambda head, and the corresponding proteins are co-expressed on the capsid surface. With such a strategy, large proteins containing up to several hundred residues could be displayed at high density [43]. 

**Figure 2:**
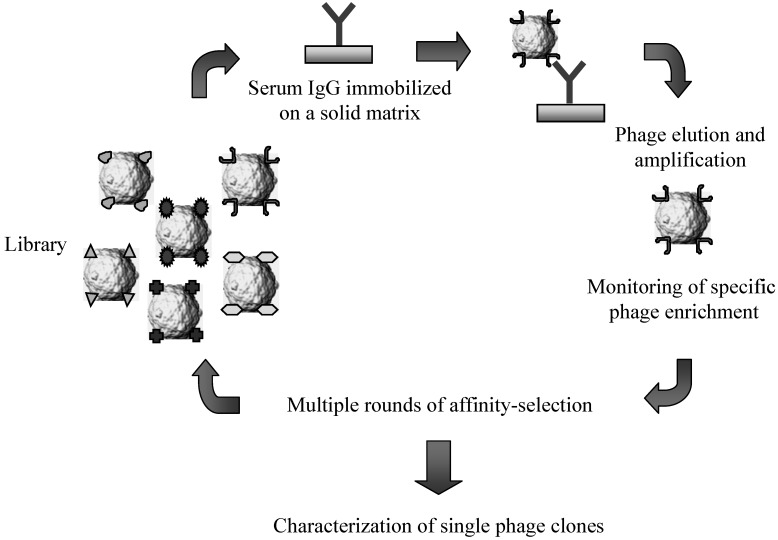
Schematic representation of the affinity-selection procedures employed for challenging lambda-display libraries of antigen fragments with serum immunoglobulins.

Within this context, several vectors have been designed to allow an efficient representation of complex mixture of coding sequences [19,44,45,46,47]. One of the most commonly used vector for displaying large genomic and cDNA repertoires with the two-gene system has a genomic copy of the *D* gene harbouring an amber mutation at the 5’ end and an additional copy which is under the control of an inducible promoter [19,45]. This vector, named λKM4, contains unique cloning sites at the 5’ end of *D* followed by a sequence coding for a flexible GS-linker between the insert and the capsid protein. Accordingly, phage particles grown on a suppressor bacterial strain display an array of wild type gpD (encoded by the genomic copy) and recombinant gpD (encoded by the additional copy) fused to foreign polypeptides. The vector also harbours an antibiotic resistance gene (*i.e.*, β-lactamase), allowing growth of recombinant clones as antibiotic-resistant lysogenic colonies.

Most of the selection schemes for screening lambda display libraries utilize a protocol which has been originally developed for filamentous phage applications [48]. Briefly, the target molecules (e.g. antibodies and protein domains), used either in solution or linked to solid-phase matrices (*i.e.*, polypropylene plates or tubes, sepharose matrices, magnetic beads), are challenged with a suspension of lambda phage particles representing the library. After incubating the mixture for several hours, phage-ligand complexes are extensively washed and then eluted under acid or basic conditions. Finally, the selected phage population is used to infect freshly cultured bacteria and the lambda phage progeny released by host cells can be directly screened for isolating individual clones or further processed for another round of affinity selection (Figure 2). Generally, the selection procedure includes 2–3 rounds of affinity selection and re-amplification of phage pools to eliminate most of the low-affinity binders and unspecific interactions.

## 4. Lambda Display for Antigen Discovery

Thus far, a large number of antigens involved in the human antibody response against infectious diseases were classically identified through the study of monoclonal antibodies elicited against gene products, utilizing either the whole pathogen or sub-fractions as immunogens. Subsequently, the genes whose products are recognized by monoclonal antibodies were isolated by the use of cDNA expression libraries, typically λ-gt11 based vectors [49], followed by expression of the corresponding protein products. Finally, recombinant proteins of the cloned genes were generally produced in bacterial cells and their immunoreactivity analyzed with sera from infected individuals. 

However, this standard procedure presents two major limitations: *(i)* bacterial cells very often do not properly fold and efficiently express long polypeptide sequences, and are not able to perform post-translational modifications which are needed to properly express many antigens from complex eukaryotic organisms such as protozoa, viruses and fungi; *(ii)* given the large number of gene products of many pathogens, it is likely that the identification of many potential antigens will fail because of the lack of specific monoclonal antibodies. 

In order to avoid these limitations, the lambda display approach has been employed to different human infectious diseases by selecting the antigenic regions harbouring B-cell epitopes via a direct challenge of pathogen’s derived protein-fragments display libraries with the whole antibody repertoire of infected individuals (*i.e.*, antibodies present in plasma or serum). 

The first example towards the use of lambda display for antigen discovery was the screening of a human hepatitis C virus (HCV) cDNA library with monoclonal antibodies or sera from HCV-infected individuals, where foreign DNA inserts were cloned into lambda genome by tagged random-primed elongation [35]. The results demonstrated that several different protein domains displayed on lambda gpD could be incorporated into viable particles and that they were accessible for interaction and affinity-selection with specific antibodies.

While HCV served as a useful test case, the question on whether cDNAs from larger genomes could adequately be represented by lambda display libraries was recently addressed in a project aimed at identifying a large panel of antigens involved in the human immune response against infection by *Toxoplasma gondii*, a worldwide distributed protozoan parasite causing significant morbidity and mortality in congenitally infected and immunocompromised individuals [50]. To this aim, several lambda display libraries of *T. gondii* cDNA fragments have been constructed and challenged with sera from either pregnant women with acquired infection or children with congenital toxoplasmosis [15,19,51,52]. The results demonstrated that a panel of lambda phage clones, displaying antigen fragments of the parasite, could be specifically enriched by using patients’ sera, and a large number of reactive phage clones were isolated. The cDNA inserts of selected recombinant phages matched the sequence of several *T. gondii* genes encoding products of different protein families such as the dense granule proteins (GRA1, GRA2, GRA3, GRA7 and GRA8), the microneme proteins (MIC2, MIC3, MIC4, M2AP and AMA1), the surface proteins (SAG1) and the bradyzoite antigens (MAG1 and BAG1).

The immunological properties of the selected antigens fragments were characterized by expressing in bacterial cells the corresponding recombinant proteins as fusion products, either at the carboxyl-terminus of GST or at the amino-terminus of lambda protein D. Most of the selected antigenic regions displayed a broad and specific B-cell immunoreactivity. The antigenicity of recombinant fragments was further investigated by looking for the presence of specific T-cell epitopes recognized by human lymphocytes. Challenging the panel of recombinant antigens with peripheral blood mononuclear cells (PBMC) from individuals with acquired or congenital *T. gondii* infection, it was demonstrated that the selected fragments induced a specific proliferative response of PBMC and secretion of pro-inflammatory cytochines, although having different frequencies in T cell activation [51,52,53].

These studies demonstrated that the use of lambda display libraries of cDNA fragments allowed the identification of a large panel of antigenic regions of *T. gondii* that are involved in both humoral and cellular immunity in humans. These results highlighted the potential of lambda display technology for antigen discovery and for the study of the human immune response against infectious agents. Moreover, the investigations validated the strategy as a general approach to study the human B-cell response against parasitic infections and other pathologies. 

A similar experimental design was successfully applied to the study of humoral immune response to another human pathogen, *Streptococcus pneumoniae* (pneumococcus), a ubiquitous gram-positive bacterium causing invasive diseases such as pneumonia, sepsis and meningitis [54,55]. In a project aimed at the identification of novel pneumococcal antigens, a lambda display library of DNA fragments from the whole bacterial genome was constructed and challenged with sera from patients hospitalized for *S. pneumoniae* diseases [16]. The affinity-selection procedure allowed the identification of several phage clones carrying *S. pneumoniae* B-cell epitopes. Epitope-containing fragments within the families of the histidine-triad proteins (PhtE, PhtD), the choline-binding proteins (PspA, CbpD) and zinc metalloproteinase B (ZmpB) were identified. Library screening also permitted the isolation of phage clones carrying three distinct antigenic regions of a hypothetical pneumococcal protein, encoded by the open reading frame *spr0075* in the R6 strain genome sequence. 

In a further study, challenging the *S. pneumoniae* lambda display library with sera from patients with acute pneumococcal pneumonia allowed the identification of an immunodominant epitope of the bacterial immunoglobulin-A protease [20], a proteolytic enzyme playing a major role in pathogen's resistance to host’s immune response. The results demonstrated that this epitope is conserved in all pneumococcus serotypes and in two out of three *S. mitis* strains, while it is not present in other oral streptococci so far sequenced. Noteworthy, the immunodominant epitope was specifically recognized by antibodies present in sera from 90% of healthy adults and 68% of children less than 4 years old, thus representing an important target of the humoral response to *S. pneumoniae* and *S. mitis* infection. 

More recently, the lambda display technology was employed for investigating the human B-cell response to *Mycoplasma pneumoniae*, a bacterial pathogen causing atypical pneumonia in children and young adults, and also involved in other severe respiratory tract diseases, such as tracheobronchitis, bronchiolitis and croups [56]. So far, gene structure and function analysis of *M. pneumoniae* have been limited by the lack of classical translational systems and the inability to express cloned genes in native *Mycoplasma* hosts. Like mitochondria, *M. pneumoniae* utilizes the UGA stop codon to encode tryptophan residues rather than serve as a termination codon [57]. Consequently, any *M. pneumoniae* gene containing one or more UGA codons cannot be expressed in expression systems that strictly adhere to the universal genetic code, due to premature termination of polypeptide chains. Because of such a peculiar characteristic, very few bacterial genes have been expressed as recombinant proteins. 

To decrease the statistical likelihood of premature termination of *Mycoplasma* proteins in *E. coli* cells, a lambda display library of the whole bacterial genome was constructed by minimizing the size of DNA fragments cloned into lambda vector (less than 200 base pairs), even if using this approach some epitopes could be missed [17]. By challenging the library with sera from patients hospitalized for *M. pneumoniae* diseases, a collection of antigen fragments corresponding to known and unknown gene products was selected [17]. Among the already known *M. pneumoniae* antigens, the immunogenicity of the adhesins P1 and P30 was confirmed. Moreover, the data presented in this study localized, within their sequences, the B-cell epitopes recognized by human immunoglobulins. Furthermore, library screening allowed the identification of four novel immunogenic polypeptides, respectively encoded by fragments of the MPN152, MPN426, MPN456 and MPN500 open reading frames, whose products were uncharacterized and, consequently, were unknown as *M. pneumoniae* antigens. All of these polypeptides were efficiently expressed and purified as native proteins from recombinant *E. coli* cells, thus further substantiating the use of lambda display for the provision of recombinant antigens.

Similarly to the above studies, the lambda display system was also applied to screen a whole genome library of the human cytomegalovirus (HCMV) for the identification of the antigenic regions recognized by immunoglobulins present in sera from HCMV infected individuals [18]. Both sera from pregnant women with acquired HCMV infection and children with congenital disease were used for the affinity-selection procedure, leading to a large collection of recombinant phages with DNA inserts matching the sequences of known antigens as well as unknown gene products. Epitope-containing fragments within the families of tegument proteins (pUL25, pUL32), structural proteins (pUL48, pUL56) and glycoproteins (pUL55) were identified. Moreover, library screening permitted isolation of phage clones carrying an antigenic region of an uncharacterized HCMV protein encoded by the UL71 open reading frame, thus further highlighting the potential of lambda display technology in antigen and epitope discovery. All peptide sequences, when removed from the lambda display context and expressed as fusion proteins, were efficiently produced in bacterial cytoplasm, thus further demonstrating that the lambda display approach easily provides a wide panel of recombinant antigens.

## 5. Clinical Applications and Perspective

The identification of pathogen-derived antigens by the direct challenge of lambda display libraries with sera from infected individuals has demonstrated to be a very powerful approach, providing novel reagents potentially very useful in different applications. The results obtained by the use of lambda display for antigen discovery are summarized in Table 1 and some valuable applications are further discussed below using the *T. gondii* project as a model system. In particular, the experimental work performed on human toxoplasmosis is used here to illustrate the potential of lambda display in the diagnostic field and for identifying antigens useful as targets for vaccine development. 

**Table 1:** Use of lambda-display libraries for antigen discovery and diagnostic applications.

Organism	Applications	Libraries	Results	References
*Toxoplasma gondii*	Antigen discovery	Whole parasite cDNA and stage-specific gene collections	Identification of a large panel of antigens containing B- and T-cell epitopes	[38,46,47]
*Toxoplasma gondii*	Development of diagnostic immunoassays	Whole parasite cDNA and stage-specific gene collections	Assay prototypes for diagnosis of congenital toxoplasmosis in pregnant women and infants	[63,64,65]
*Toxoplasma gondii*	Development of DNA-based vaccines	Stage-specific gene collections	DNA vaccines conferring protective immunity against chronic toxoplasmosis	[48,66,68]
*Streptococcus pneumoniae*	Antigen discovery for vaccine development	Genomic DNA	Identification of a large panel of immunodominant antigens	[52]
*Streptococcus pneumoniae*	Epitope mapping	Genomic DNA	Isolation and characterization of streptococci conserved epitopes	[53]
*Mycoplasma pneumoniae*	Antigen discovery for diagnostic applications	Genomic DNA	Isolation and characterization of B-cell regions for diagnostic immunoassays	[56,69]
Human cytomegalovirus (HCMV)	Antigen discovery	Genomic DNA	Identification of a large panel of B-cell epitopes	[57]
Human Hepatis C Virus (HCV)	Antigen discovery	Whole viral cDNA	Molecular dissection of the B-cell response	[29]

As mentioned above, infection with *T. gondii* causes morbidity and mortality in congenitally infected and immunocompromised individuals. Toxoplasmosis during gestation represents a formidable task for the clinician due to its subclinical course in the majority of pregnant women and the unpredictable long-term outcome of congenital infection [58,59,60]. To implement suitable therapies in good time and to avoid complications in newborns, it is crucial establishing when the primary infection has been acquired in the mother and determining if vertical transmission to the foetus has occurred. 

Diagnosis of primary *T. gondii* infection depends on detection of Toxoplasma-specific IgG and IgM antibodies but does not make it possible to estimate the time of infection with accuracy [61,62]. Measurement of specific IgG avidity has been shown to be the best tool, in combination with IgM analysis, for determining when the infection took place. In this context, the antigenic regions selected by lambda display have been used to develop antibody-specific IgG-avidity assays for discriminating between acute and latent phases of *T. gondii* infection [63]. In a retrospective study, a large panel of serum samples from women who developed IgG antibodies against *Toxoplasma* during pregnancy was used. The IgG avidities of antibodies directed against epitopes carried by fragments of GRA3, GRA7, MIC3, and SAG1 antigens were measured by performing parallel enzyme immunoassays. The MIC3 antigen fragment was found to be a good marker for diagnosing recently acquired infections, and both high- and low-avidity results could be used to determine the time of infection. This is important to know when pregnant women ask for a test of immunity, which in most countries is usually performed between gestational weeks 8 and 14. Therefore, the results obtained by the avidity assay with recombinant antigens could be successfully applied for the diagnosis of the acute phase of *T. gondii* infection during pregnancy. 

The clinical usefulness of recombinant antigens selected by lambda display has also been investigated in a study aimed at improving the early serologic diagnosis of toxoplasmosis in children at risk of congenital infection. To this aim, the immunoreactivity of seven distinct fragments of *T. gondii* (MIC2, MIC3, MIC4, M2AP, AMA1, and SAG1 fragments), expressed as GST fusion products, was assessed with IgM, IgG, and IgG-subtype antibodies present in sera from infants born to mothers with primary *T. gondii* infection in pregnancy [64]. Recombinant antigens preferentially reacted with IgG antibodies from infected infants compared to uninfected subjects, indicating that sera from infected children recognized a more diverse repertoire of antigens than sera transferred over the placenta from the mothers. Moreover, it was possible to demonstrate an early neosynthesis of anti-MIC2 and anti-SAG1 IgG (within 3 months of life) in sera from infants with congenital infection. Finally, IgM antibodies in 97% of infected infants reacted with at least one of the recombinant antigens, confirming the diagnosis of congenital infection as soon as 2 months after birth. Thus, this study clearly shows that assays based on antigen fragments selected by lambda display improve the diagnosis of newborns with congenital toxoplasmosis. 

Finally, a more elaborated approach was employed to evaluate better the diagnostic utility of six antigen fragments selected by lambda display (MIC2, MIC3, M2AP, GRA3, GRA7, and SAG1) and further assembled in recombinant chimeric antigens by genetic engineering [65]. By using a standard bacterial expression system, the chimeric antigens were purified under native conditions in large amounts and were found to have good solubility, excellent thermal stability and elevated purification yields, which are fundamental prerequisites for the commercial use of a recombinant product. Overall, the study demonstrated that assays employing the chimeric antigens have performance characteristics comparable to or even better than those of assays that use the whole-cell parasite extracts, thus indicating that the chimeric antigens could provide the basis for standardized commercial immunoassays for the serodiagnosis of toxoplasmosis.

Development of vaccines against *T. gondii* infection in humans is of high priority, given the high disease burden and the lack of effective drugs with few adverse effects. In the last decade, efforts have been made to develop an anti-Toxoplasma vaccine, whose feasibility was suggested by the long-term immunity induced by the primary infection. However, regardless of the vaccine construct, the vaccines have not been able to induce sterile immunity when the organism is challenged with *T. gondii*, either directly or via a vector. 

Within this framework, several studies have been conducted enrolling the antigen fragments selected by lambda display in the design of recombinant antigens- and DNA-based vectors to be used as anti-Toxoplasma vaccines [52,66,67,68]. 

In the first study, the ability of the selected microneme fragments (protein fragments released from secretory organelles, the micronemes, during parasite invasion) of *T. gondii* to induce, in a DNA-vaccine formulation, an effective protective immunity in mice against chronic toxoplasmosis was evaluated [52]. To this purpose, the DNA encoding six microneme fragments (MIC2a, MIC2b, MIC3, MIC4, M2AP, and AMA1) were separately cloned into a mammalian expression vector, and a homogeneous mixture of the corresponding plasmids was used to immunize the mice. The protective immunity induced by DNA immunization was evaluated by orally infecting the vaccinated mice with 30 cysts of an avirulent *T. gondii* strain. The results showed that DNA immunization with microneme fragments elicited effective protection in mice (84% reduction in brain-cyst burden), strongly indicating that a combination of these antigenic regions should be considered in the design of vaccines against toxoplasmosis.

A similar study using a combination of antigens delivered as plasmids coding for two bradyzoite (cyst-specific) fragments (BAG1 and MAG1) in a cocktail DNA vaccine also showed a significant reduction in the number of brain cysts (62% reduction compared with animals vaccinated with empty vector) [66], suggesting that bradyzoite antigens should also be included in a poly-component vaccine to prevent *T. gondii* infection.

**Figure 3:**
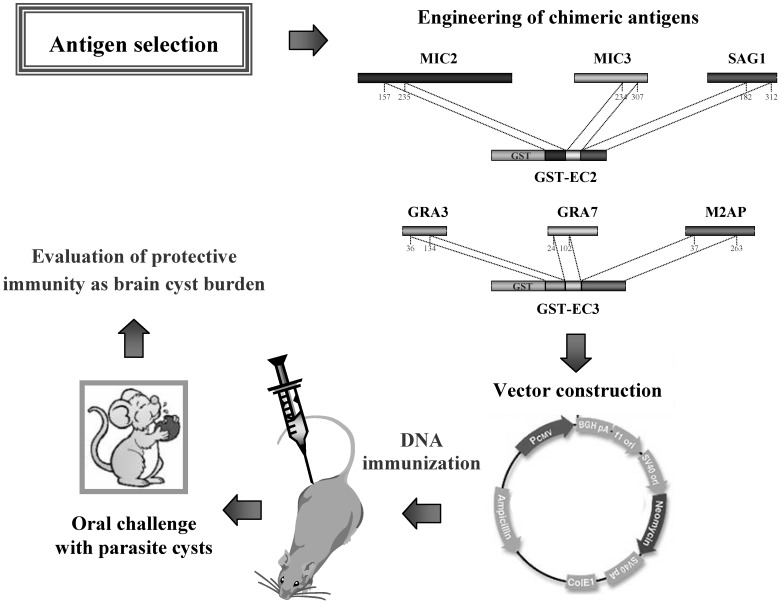
Engineering of chimeric antigens for DNA vaccine formulations used in animals against infection by *Toxoplasma gondii*.

The use of chimeric antigens in a vaccine formulation should be an advantage, theoretically, giving the opportunity of including multiple epitopes in a common polypeptide sequence. Accordingly, a recent study evaluated the protective immunity elicited by DNA immunization employing two *T. gondii* chimeric antigens (EC2 and EC3), which were previously constructed for diagnostic applications [65]. In Figure 3 is schematically shown the design of this study. Overall, the results demonstrated that mice vaccinated with the chimeric DNA vaccines and then challenged with a low-virulent *T. gondii* strain survived infection [68]. In particular, immunisation with pDNA-EC2 or pDNA-EC3 lead to 84.7% and 93.5% reduction of brain cyst load, respectively, compared to the animals vaccinated with empty vector, thus further supporting the use of antigen fragments and/or immunodominant epitopes selected and characterized using the lambda display technology in vaccine applications.

## 6. Conclusions

Although phage display technology was introduced twenty years ago, the possible applications of this biological approach have been continuously in practice. Exploitation of phage display has lead to the isolation and production of a broad range of molecules, including recombinant antibodies and antigens, with predefined specificity.

**Figure 4:**
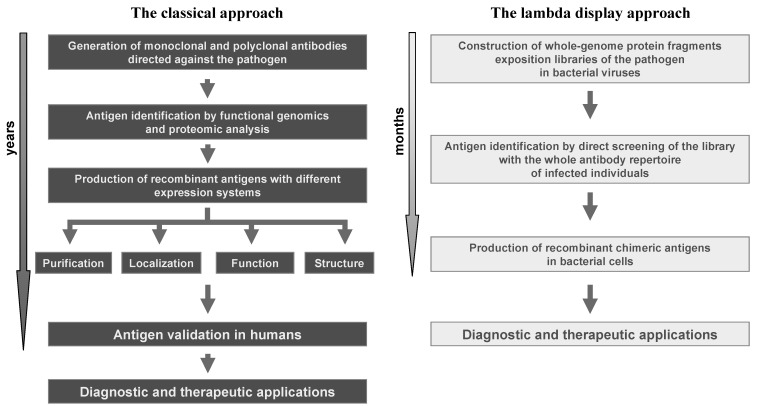
Comparison of the lambda display approach with respect to classical methods in antigen discovery applications.

The experimental successes achieved by the use of lambda display strongly confirm the great value of this technology in studying protein-protein interactions and, particularly, in antigen discovery. With respect to classical technologies, the lambda display approach gives a high chance to identify large panels of antigenic regions and immunodominant epitopes in a short period of time and at very low cost, and it is a powerful method for identifying sequences from large repertoires such as those derived from libraries of cDNA or whole genomes (*i.e.*, viruses and prokaryotes). Moreover, lambda display libraries can be easily screened against complex mixtures, such as antibodies present in sera from infected individuals. Finally, the antigenic regions characterised by lambda display may constitute powerful leads for improving diagnosis, prophylaxis and therapy of life-threatening diseases induced by human pathogens (Figure 4).
